# Comparative Study of Ni-Impregnated Alumina Aerogels and Ni-Al Xerogels for Light-Irradiation-Assisted CO_2_ Methanation

**DOI:** 10.3390/gels12050420

**Published:** 2026-05-11

**Authors:** Daniel Estevez, Haritz Etxeberria, Victoria Laura Barrio

**Affiliations:** School of Engineering of Bilbao, University of the Basque Country (UPV/EHU), Plaza Ingeniero Torres Quevedo 1, 48013 Bilbao, Spain; daniel.estevez@ehu.eus (D.E.); haritz.echeverria@ehu.eus (H.E.)

**Keywords:** CO_2_ methanation, Sabatier reaction, power-to-gas, greenhouse gas emissions, nickel, alumina, xerogel, aerogel, light-assisted CO_2_ methanation

## Abstract

CO_2_ methanation is considered a key process in achieving carbon neutrality. Expanding on our previous study of supercritically dried Ni-Al aerogels, this work compares two gel-based catalyst families prepared via two different routes—supercritically dried Ni impregnated Al aerogel-based catalysts and oven-dried one-pot Ni-Al xerogel-based catalysts—to assess how the synthesis route affects catalyst structure and CO_2_ methanation performance under light irradiation. The catalysts were subsequently characterized via different techniques, such as ICP-OES, N_2_ adsorption–desorption isotherms, XRD, H_2_-TPR, UV-vis DRS, XPS, and TEM. Catalytic activity was tested in a photoreactor at a range of temperatures from 300 °C to 450 °C and 10 bar pressure, and two different light sources were used (λ = 365 nm, λ = 470 nm). Both light sources enhanced catalytic activity in most cases; the xerogels with higher Ni loadings were the most active materials. These catalysts reached CO_2_ conversions and CH_4_ selectivities near 70% and 100%, respectively. The results indicate that drying gels is a promising method for synthesizing catalysts active in the Sabatier reaction, given the properties of the materials.

## 1. Introduction

The core objective of the European Green Deal (EGD), launched in 2019, is to achieve climate neutrality in the EU by 2050 by eliminating net greenhouse gas emissions. This reduction in emissions not only helps protect the planet but also saves human lives, as it relates to different conditions such as ischemic heart disease and lung cancer [1]. The approach of the EGD in reducing these emissions is to address the main sources of pollution. These include the agriculture, industry, transport, and power generation sectors. In 2024, agricultural activities emitted 363 million tons of CO_2_ into the atmosphere [2]. The European Union has encouraged the use of organic farming, reducing the use of fertilizers by 20%, as well as chemical pesticides by 50%, which are responsible for a large share of the agricultural emissions. This measure, together with the rotation of crops, composting, and natural pest practices, should significantly reduce carbon emissions [3]. The transportation sector has been compelled to reduce carbon emissions by 37.5% by 2030 to lower its current contribution of 20% to global greenhouse gas emissions. This will be achieved by electrifying the different means of transportation [4]. From the point of view of the power sector, the EGD focuses on developing sustainable and competitive systems based on different decarbonization routes, such as renewable energies and carbon capture and storage technologies [5]. To achieve a 100% renewable power system by 2050, several objectives need to be accomplished. Some of these objectives are to expand the energy generation capacity to almost double its current level, integrate electric vehicles via smart charging and other technologies, and implement energy efficiency systems to prevent large increases in electrical demand [6].

Renewable energies are vital to achieving sustainable development and drastically reducing greenhouse gas emissions, and given their increasing affordability [7], they are the most promising alternative to power generated from traditional sources. However, one of the downsides of these technologies is the lack of renewable energy input at certain times, when backup energy is needed. Gaseous fuels represent an attractive option for energy storage due to their high energy density [8], and one of the most common ways to convert this energy into molecules is through power-to-gas (PtG) technologies, which include the commonly used process of CO_2_ methanation. Discovered by Paul Sabatier in 1902, it is an exothermic reaction in which CO_2_ is converted into CH_4_ in the presence of a catalyst and at high temperature [9]. The Sabatier reaction (1) can be described as the combination of two different reactions [10]. The first one (2) is the reverse water–gas shift reaction (RWGS), where CO_2_ reacts with H_2_ to form CO and H_2_O. Afterwards, the CO produced reacts with additional H_2_ (3) and finally generates CH_4_, along with another water molecule.

(1) $${CO}_{2}+4H_{2}↔{CH}_{4}+2H_{2}O\;∆H^{o}=-165.2\;KJ\;{mol}^{-1}$$

(2) $${CO}_{2}+H_{2}↔CO+H_{2}O\;∆H^{o}=+41.1\;KJ\;{mol}^{-1}$$

(3) $$CO+3H_{2}↔{CH}_{4}+H_{2}O\;∆H^{o}=-206.3\;KJ\;{mol}^{-1}$$

The original reaction converts CO_2_ into CH_4_ at a very low rate due to kinetic limitations arising from the high stability of the CO_2_ molecule and the energy barrier for hydrogen activation [11]. To enhance the overall efficiency, the development of catalysts with photocatalytic activity has been a widely studied topic in recent years [12,13,14]. For instance, Zhu et al. developed Ni-TiO_2_-based catalysts that demonstrated catalytic activity 2.7 times higher under a 1200 mW cm^−2^ light source compared to dark conditions [15]. The search for suitable catalysts, in terms of active metal and support, remains an ongoing challenge for researchers.

In the case of active metals, noble metal-based and non-noble metal-based catalysts have been developed. Regarding noble metal-based catalysts, some of the most commonly used elements are rhodium [16,17,18], ruthenium [19,20,21] and platinum [22,23]. In the case of Rh, CO_2_ conversions near 90% and methane selectivity above 90% were obtained at 350 °C with an Al_2_O_3_-supported Rh catalyst [24]. Ru usually exhibits higher activity than Rh in the CO_2_ methanation reaction, as shown in a study where 82% conversion of CO_2_ and almost 100% of CH_4_ selectivity were obtained at 250 °C [25]. Pt, unlike Ru and Rh, stands out for its high selectivity towards CH_4_ rather than its CO_2_ conversion in the Sabatier reaction [26]. This is why it is usually used as a co-catalyst or promoter, as shown in a study where Pt was added to a monometallic Ni/TiO_2_ catalyst, improving methane selectivity to nearly 100% across the whole temperature range [27]. Noble metals are expensive due to their scarcity, which makes them industrially challenging; thus, cheaper and more available metals are usually employed as catalysts. These metals are usually Ni [28,29,30], Fe [31,32,33] and Co [34,35,36]. In terms of activity, Fe presents better performance than Ni, followed by Co, whereas Ni exhibits the highest CH_4_ selectivity of the three [37]. Ni is a widely used metal in the CO_2_ methanation reaction, given its overall good performance, as illustrated by a Ni/CeO_2_ catalyst synthesized via the wet impregnation method. In this work, the catalyst achieved a CO_2_ conversion of 84% and a methane selectivity near 100% at 300 °C and atmospheric pressure, which are quite mild conditions [38]. Even if the overall efficiency of the Ni catalysts is high, they present a few drawbacks related to deactivation. First, they suffer from sintering caused by high temperatures, which reduces the specific surface area. Secondly, they can be poisoned by compounds containing S, P, or NH_3_ that are usually present in the feed stream. Lastly, the active sites of the catalyst can be blocked by carbon deposition, which reduces activity [39].

To reduce energy consumption, mitigate these deactivation mechanisms, and tune the surface properties of the catalysts, such as surface area, active metal dispersion, or acidity, selecting an adequate support is mandatory in the synthesis of these materials. Typically, different metal oxides have been used as supports, including Al_2_O_3_, CeO_2,_ and ZrO_2_ [40]. Depending on the material chosen, the support can act as a promoter, as in the case of CeO_2_ due to Ni-O-Ce interactions [41]; as a co-catalyst, as reported for ZrO_2_ [42]; or just as a high-surface-area carrier that enables high metal dispersion, as in the case of the alumina [43].

To address the need for new catalytic systems with enhanced surface properties and stability, new kinds of support were adopted from already existing materials. For instance, gel-based supports can be developed through various drying methods: evaporating the solvent via supercritical drying, conventional evaporation of the solvent, and freeze-drying [44]. In the case of the latter, the gels are frozen at an adequate temperature and freezing time, depending on the solvent, which is later extracted by thawing [45]. The result of this process is called cryogel. The thermal evaporation of the solvent produces the so-called xerogels. This process is the most aggressive one, as the fast extraction of the solvent generates capillary forces that can provoke the collapse of the pores in the gel structure [46]. To avoid this drawback, the supercritical drying of the gels has been used as a method to maintain the network structure. In this case, the solvent is extracted from the gel by soaking it in liquid CO_2_ or acetone, which is later turned into a supercritical state, which reduces the mechanical stress on the pore walls [47]. The results of those three processes are three-dimensional materials that maintain the structure of the original gel. These dried gels are typically very porous, stable, and high-surface-area materials, which allow active metals to properly disperse when used as catalytic support. Among the three of them, aerogels are the most appropriate to use as support, as the lack of capillary stress avoids the collapse of the pores and thus, the surface area is superior to the rest of the drying methods. Supercritical drying needs to reach high pressures, which means a high energy demand, resulting in the need for complex equipment [48]. Therefore, prior to performing the gel drying, the most suitable of the three methods must be chosen, according to the requirements of the final product.

This work aims to continue the work of previous studies [49]. While in previous works, the feasibility of supercritically dried Ni-Al aerogels for CO_2_ methanation was established, the present study addresses a different question: how do two gel-based catalyst families prepared through distinct routes differ in Ni incorporation, phase composition, reducibility, and catalytic response under identical reaction conditions? Al-based aerogels (AGs) were synthesized and subsequently dried via supercritical CO_2_ drying, followed by the impregnation of Ni salt. Then, oven-dried xerogels (XGs) with different Al/Ni ratios were prepared. Both types of catalysts were characterized using different simple and advanced techniques: N_2_ adsorption–desorption isotherms, inductively coupled plasma optical emission spectroscopy (ICP-OES), transmission electron microscopy (TEM), powder X-ray diffraction (XRD), X-ray photoelectron spectroscopy (XPS), ultraviolet–visible diffuse reflectance spectroscopy (UV-vis DRS), and hydrogen temperature-programmed reduction (H_2_-TPR). These results presented a comprehensive overview of the physicochemical, structural, and compositional properties of the developed catalysts. The catalytic activity under dark and irradiated conditions of CO_2_ methanation was measured in a bench-scale plant with an 8 mm open and removable quartz window, which had an attached LED light source. The reactions were carried out over a temperature range of 300 to 450 °C, at a constant pressure of 10 bar, using either no light, a 470 nm light source, or a 365 nm light source as activity enhancers. The catalytic mass to perform each reaction was 5 mg. The characterization and catalytic activity results of this study, together with those from the previous article, provide a broader understanding of the impact of different synthesis and drying methods on gel-based catalysts.

## 2. Results and Discussion

### 2.1. Textural Properties

Table 1 shows the Al/Ni molar ratios of the different catalysts, obtained via ICP-OES. Moreover, Table 1 also shows the results, where S_BET_, V_t,pore_, and D_BJH_ refer to the surface area calculated using the Brunauer–Emmett–Teller (BET) method, the total pore volume, and the pore diameter obtained using the Barrett–Joyner–Halenda (BJH) model, respectively.

In the case of the xerogels, the molar ratios were slightly below the theoretical value, whereas the ones corresponding to the impregnated aerogels were significantly lower, due to a loss of support mass during the impregnation process. Regarding the surface properties, the impregnation reduced the specific surface area of the catalysts, compared to the non-impregnated Al aerogel, as expected. The impregnated AGs presented lower pore volume and particle diameter values than the base support, which might be attributed to the impregnation [50]. In the case of the xerogels, a clear correlation is observed: as the Al/Ni molar ratio increases from 0.33 to 4.00, the specific surface area improves significantly (from 135 to 276 m^2^/g). This indicates that a higher aluminum content plays a crucial role in maintaining a more open and accessible porous structure, which may be beneficial for mass transfer during catalysis.

For all the catalysts, the isotherms correspond to Type IV isotherms, which are characteristic of mesoporous materials. These are shown in Figure S1 of the Supplementary Material.

### 2.2. X-Ray Diffraction

**Figure 1:**
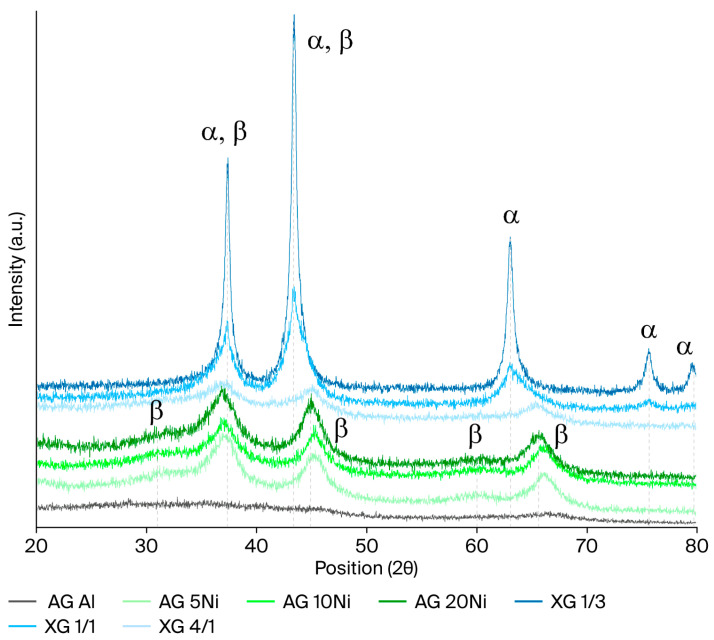
XRD profiles of xerogels and impregnated aerogels. α signals correspond to NiO, whereas β signals correspond to NiAl_2_O_4_ spinel.

The X-ray diffraction results reported in Figure 1 describe the crystallinity of the different catalysts. As can be observed, two different peaks can be distinguished. The α signals correspond to NiO (PDF 00-001-1239), whereas β signals appear as a result of a minor presence of NiAl_2_O_4_ (PDF 00-001-1299). In the case of the impregnated aerogels and in XG 4/1, a higher contribution of the aluminum spinel was measured, while the xerogels with higher Ni content present a major NiO peak, as corroborated by the position and shape of some of the peaks. The presence of Al_2_O_3_ was considered; however, since none of the diffraction peaks of the sample matched those of the oxide, this possibility was discarded. Thus, the support was concluded to be amorphous. Additionally, the sample with the lowest Al/Ni ratio (XG 1/3) was the one with the highest crystallinity.

Moreover, the crystallite size was calculated using the Scherrer equation. In the case of the impregnated aerogels, the three samples (AG Al + 5Ni, 10Ni, and 20Ni) had crystallites around 4.0 nm. Regarding xerogels, samples with lower Ni content, such as XG 4/1 and XG 1/1, present similar values to the aerogels, whereas XG 1/3 presented a crystallite size near 20 nm. This higher value could be attributed to the higher crystallinity of the sample, as observed in the XRD profile.

### 2.3. H_2_ Temperature-Programmed Reduction (H_2_-TPR)

**Figure 2:**
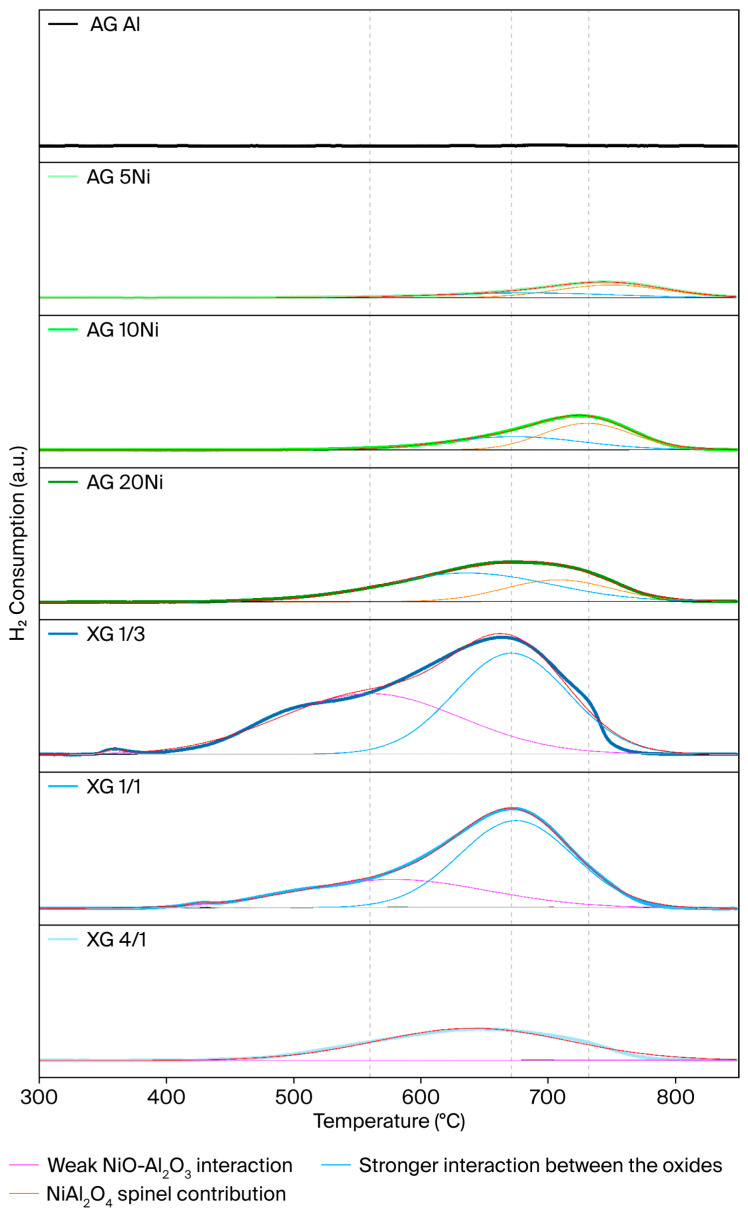
H_2_-TPR profiles of XGs and impregnated AGs. The lines under the profiles indicate the different contributions of the species present in the samples: the purple line corresponds to the weak NiO-Al_2_O_3_ interaction, the blue line corresponds to a stronger interaction between the oxides, and the orange line corresponds to the NiAl_2_O_4_ spinel contribution.

The H_2_-TPR profiles of the xerogels and impregnated aerogels are presented in Figure 2. Similar behavior was observed in both types of samples, so XG 1/3 was used as the reference to describe the different reduction peaks. Three different sections were observed in the reduction profiles, which arise from different interactions between Ni species and the support. The region at lower temperatures, which is extended up to 560 °C, was attributed to the NiO species with weak interaction with the support. When the temperature reaches 670 °C, another reduction peak appears, corresponding to the NiO-Al_2_O_3_ strong interactions [51,52]. Finally, in the case of the impregnated aerogels, one more peak appears at 730 °C. This peak is due to the presence of the NiAl_2_O_4_ spinel species, which are formed when Ni^2+^ ions are diffused into the tetrahedral space of the Al_2_O_3_ support [53,54,55]. H_2_-TPR provides evidence of interacting Ni-Al oxide species, which are consistent with NiO and NiAl_2_O_4_ spinel, while the obtained XRD results provide a more direct structural support for this assignment. It is also noted that the species with the lower Al/Ni molar ratio are the most reducible samples.

### 2.4. Transmission Electron Microscopy (TEM)

**Figure 3:**
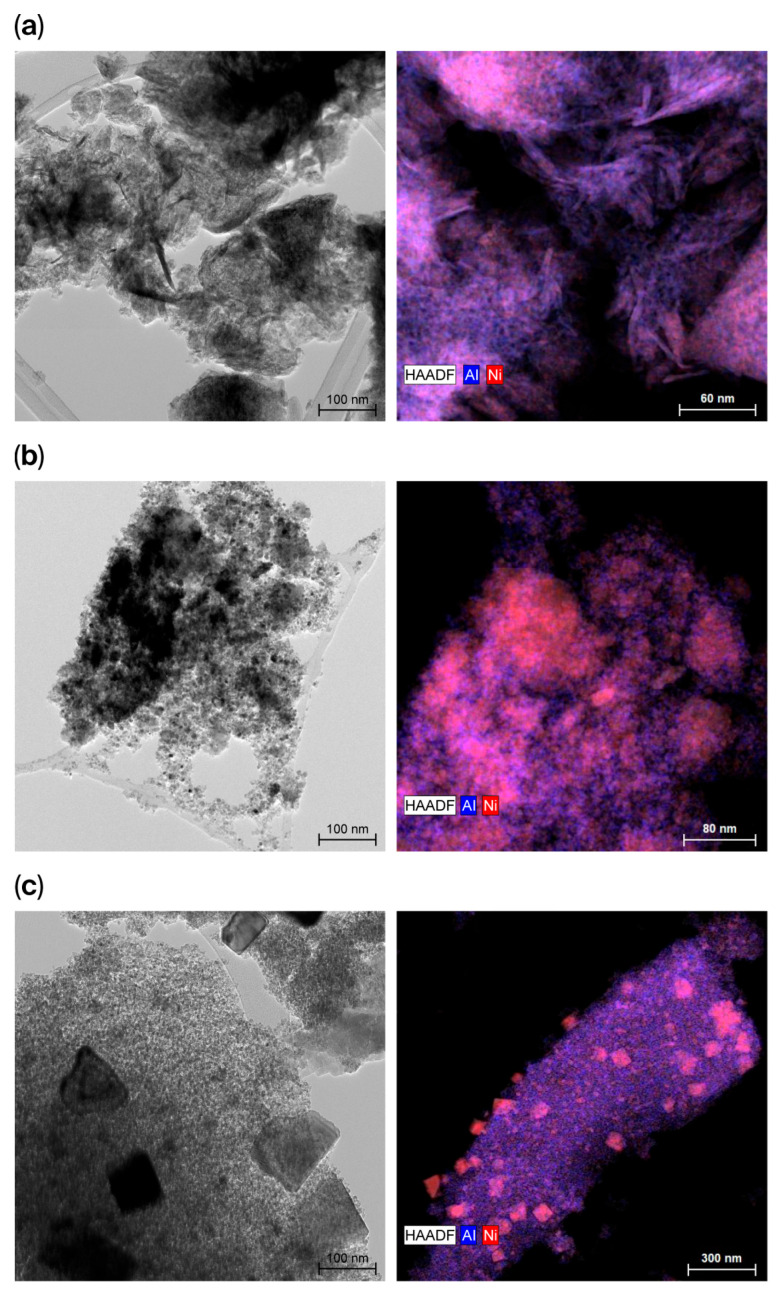
TEM micrographs (**left**) and EDX mapping (**right**) of (**a**) AG Al + 20Ni, (**b**) XG 1/3, and (**c**) XG 1/1.

The morphology of the samples was studied through TEM. It was observed that most of the samples presented an oval-like morphology. In accordance with the XRD results, XG 1/3 tended to grow hexagonal-shaped nanoparticles, as it is the sample with the highest crystallinity. The particle size of every sample containing nickel was measured. Approximately 100 particles were measured in each catalyst, and in most of the cases, particle sizes in a range between 2.5 and 6.5 nm were obtained. The aluminum aerogel, however, presented larger particle sizes, ranging from 9 to 17 nm. The smaller particle sizes observed in the Ni-containing samples may suggest that the presence of Ni species influences the nucleation and growth of the oxide network during synthesis. This effect could limit particle growth and lead to smaller nanostructures compared with the pure alumina aerogel. Additionally, the EDX maps revealed that in most of the catalysts, the Ni dispersion was adequate and homogeneous. In the case of XG 1/1, however, some spots of agglomerated Ni particles were observed, though they did not affect surface and textural properties, as seen in Table 1. A selection of catalysts is displayed in Figure 3, where the different morphological properties can be observed. The TEM micrographs for the rest of the catalysts are shown in Figure S2 of the Supplementary Material.

### 2.5. UV–Visible Diffuse Reflectance Spectroscopy (UV-Vis DRS)

**Figure 4:**
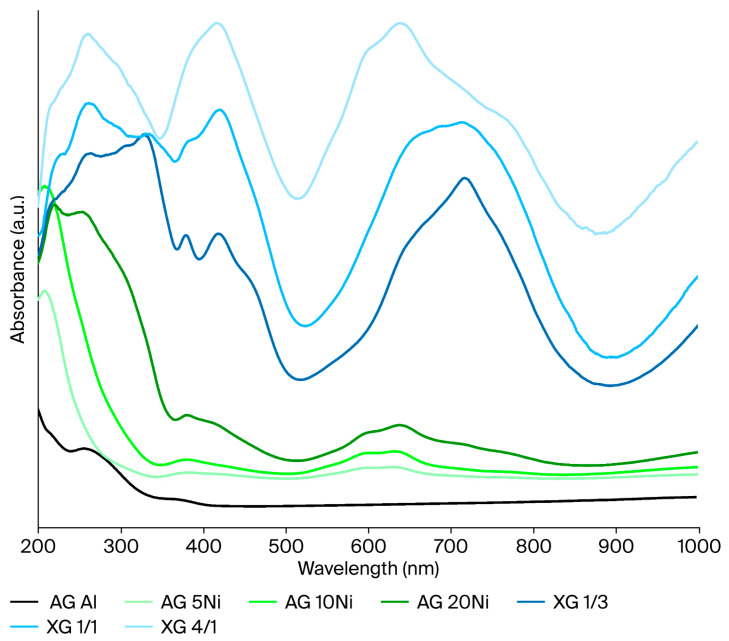
UV–visible DRS absorption spectra of the XGs and impregnated AGs.

The UV-vis DRS spectra of the samples are shown in Figure 4. As can be observed, the bands corresponding to the xerogels present a much higher absorption in all wavelengths. The absorption peak at 200–350 nm is attributed to the O^2−^ → Ni^2+^ charge transfer [56], while the following one at 350–500 nm is a consequence of the octahedral Ni^2+^ present in the NiO and NiAl_2_O_4_ species [57]. An intense and broad additional peak appears at 500–800 nm. This band is the result of the overlapping of two different peaks. The first one reaches 750 nm and corresponds to the tetrahedral Ni^2+^ of the NiAl_2_O_4_ spinel [58]. The second one appears as a shoulder, and it appears because of the presence of the d-d transition of octahedral Ni^2+^, attributed to the NiO species [59]. Moreover, it can be noted that the intensity of the spinel band increases with the Ni content.

The direct band gap value was also calculated using the Tauc equation, plotting (αhν)^2^ vs. (hν) and extrapolating straight lines of (αhν)^2^ to the *x*-axis. The Tauc plot of the catalysts is shown in Figure S3 of the Supplementary Material, and the calculated band gap energies are shown in Table 2. A decrease in the band gap energy can be observed when the Ni content increases for all the catalytic samples. The decrease in the band gap with the increasing Ni content can be attributed to the introduction of Ni-related electronic states, which shift the absorption onset to lower energies [60].

### 2.6. X-Ray Photoelectron Microscopy (XPS)

Table 3 presents the surface composition of the catalysts, obtained via X-ray photoelectron spectroscopy (XPS). In the case of the impregnated aerogels, only the one with the lowest Ni loading presented an Al/Ni ratio larger than that obtained via ICP-OES. This difference indicates that part of the Ni was distributed in the bulk and not only on the surface of the catalyst. Regarding the xerogels, only XG 1/3 presented a surface Al/Ni atomic ratio greater than the one on the bulk. In this case, this could be explained by the synthesis method, which, added to the high nickel content, could have caused the immobilization of the metal inside the structure and not just on the surface of the catalyst.

**Figure 5:**
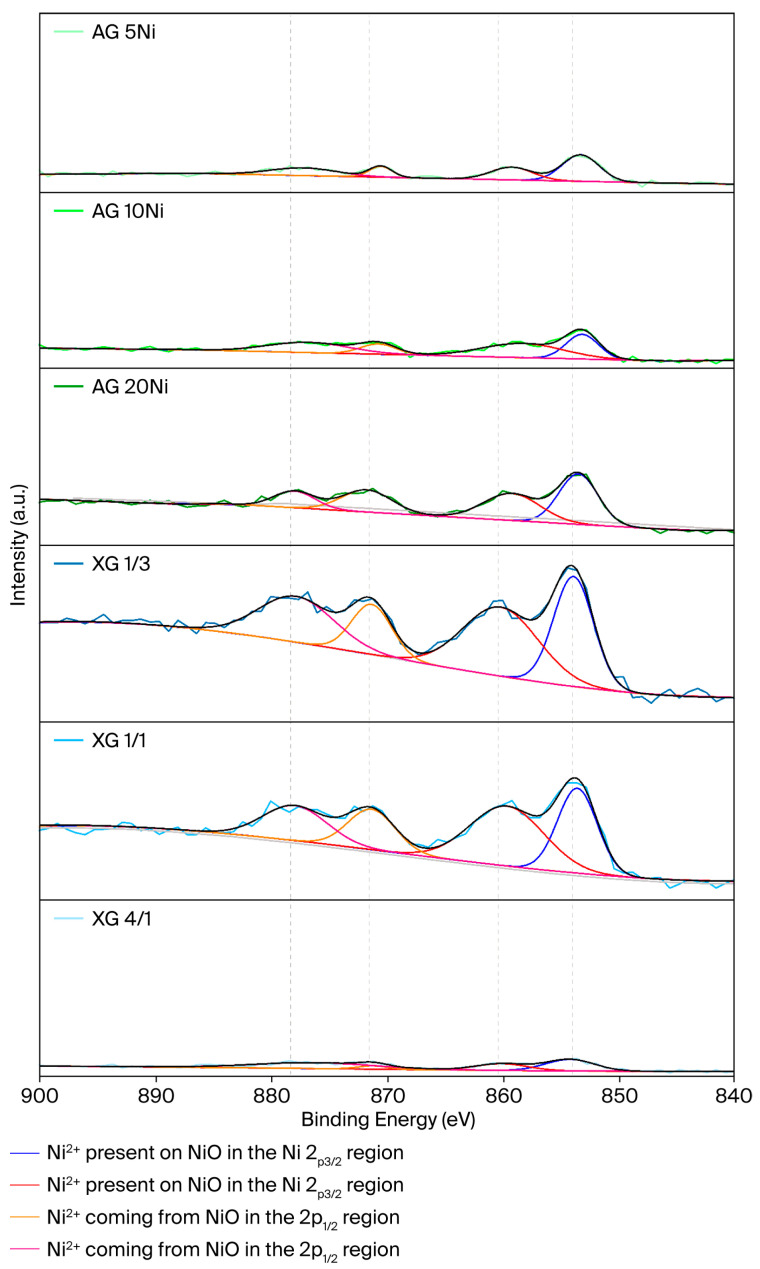
XPS spectra of the xerogels and impregnated aerogels. The lines under the signal correspond to the signals of each peak: dark blue and red correspond to the Ni^2+^ present on NiO in the Ni 2p_3/2_ region; orange and pink correspond to Ni^2+^ coming from NiO in the 2p_1/2_ region.

Figure 5 depicts the Ni 2p XPS spectra of the catalysts. The difference in the intensity of the peaks comes from the difference in the nickel content of each sample. Two different peaks appeared in each of the Ni 2p_3/2_ and Ni 2p_1/2_ sections. The Ni 2p_3/2_ region appeared to have two peaks, which had their maximums at 854.0 eV and 860.4 eV, respectively. The same behavior was found in the Ni 2p_1/2_ region, where the maximum values were found at 871.5 eV and 878.3 eV. The peaks found at these binding energies are attributed to the Ni^2+^ species found in the samples and their satellites [61,62].

In the case of the Al 2p peak, it appeared in all samples at binding energies around 74.0 eV, which is attributed to the presence of Al^3+^ ions. These ions can be part of the Al_2_O_3_ species or part of the NiAl_2_O_4_ spinel [63,64]. Regarding the O 1s spectra, only one peak appears around 531.0 eV, and it is probably the result of a combination of the contribution of the O^2−^ in the NiO species [65,66] and the contribution of the oxygen present in the NiAl_2_O_4_ spinel [67,68]. Even if the presence of the mentioned peaks confirms the existence of the Ni oxide and spinel, the proximity of the signals makes it difficult to assign each one of them to one specific species.

### 2.7. CO_2_ Methanation Activity Performance

**Figure 6:**
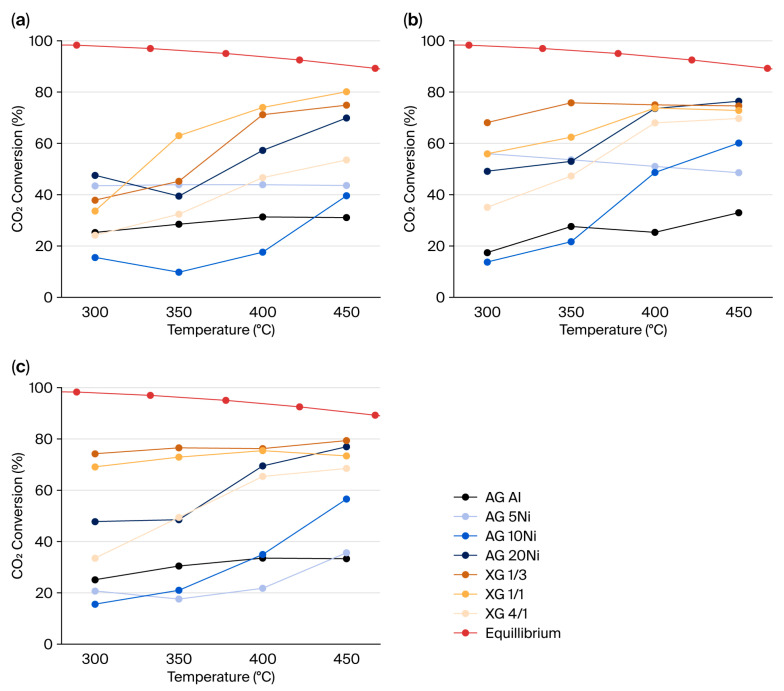
Catalytic activity under dark and irradiated conditions of xerogels and impregnated aerogels: (**a**) dark conditions; (**b**) visible light (λ = 470 nm) enhanced; and (**c**) UV light (λ = 365 nm) enhanced.

The results of the catalytic activity of the different materials are displayed in Figure 6 in terms of CO_2_ conversion.

In the case of dark conditions, high temperatures (450 °C) resulted in up to 80% of CO_2_ conversion. Catalysts with a higher Ni content, such as XG 1/3, XG 1/1, and AG Al + 20Ni, yielded the best results at higher temperatures, while the ones with lower loadings (i.e., XG 4/1, AG Al + 5Ni, and AG Al + 10Ni) reached lower values at the same temperature conditions. This behavior could be explained by the lack of nickel and the low reducibility of the catalyst, as seen in the H_2_-TPR results. Regarding CH_4_ selectivity, the catalysts with the best CO_2_ conversion also exhibited the best results, reaching values close to 100%, while the rest of them obtained lower selectivity values. Moreover, the aluminum-only aerogel did not demonstrate any significant activity, as its maximum CO_2_ conversion and CH_4_ selectivity were around 30% and 20%, respectively, at all temperatures.

When applying visible light to enhance the activity, the catalysts with higher nickel content, namely XG 1/1, XG 1/3, and AG Al + 20Ni, reached a similar CO_2_ conversion maximum value at 450 °C, but the values at the lower temperatures improved. Such is the case of XG 1/3, which obtained 68% of conversion at 300 °C in comparison to the 38% obtained in the dark conditions, or XG 1/1, which improved CO_2_ conversion from 34% to 56% at the same temperature. In the case of the catalysts with a lower metal loading (XG 4/1, AG Al + 10Ni, and AG Al + 5Ni), the activation of these materials was improved at higher temperatures than the previous ones. For example, at 400 °C, AG Al + 10Ni reached a conversion of 49%, in comparison to the 18% obtained with no light enhancement, while the XG 4/1 catalyst obtained 68% of conversion in contrast to the 47% obtained without the light source. In regard to the CH_4_ selectivity, most of the catalysts reached values above 90% from 300 °C, improving it from the non-light situation, being the AG Al + 5Ni catalyst the only one not surpassing 55% of selectivity at any temperature. Once again, the AG Al catalyst did not render any relevant result, and it was not affected in any way by the use of the visible light source.

In the case of using ultraviolet light, the results were similar to those obtained with the visible light source in most cases. Xerogels 1/3 and 1/1 slightly improved their CO_2_ conversion at the lower temperatures, but no significant change was observed at 400 and 450 °C. The cause of the similarities between the results of UV and visible light could be attributed to the high absorbance at all wavelengths, as seen in the UV-vis DRS results. In contrast, some of the impregnated aerogels exhibited a decrease in catalytic activity under UV irradiation. For instance, AG Al + 10Ni showed a lower conversion at 400 °C compared with visible light conditions. This behavior may be related to the lower light absorption capacity of the aerogel-based catalysts and to the presence of NiAl_2_O_4_ spinel species, which are harder to reduce and therefore less active in the methanation reaction. Therefore, UV irradiation does not significantly enhance the catalytic performance of these materials and may even lead to slightly lower conversions compared with visible light conditions. The selectivity towards methane was also similar to the values obtained by using the visible light source, yielding values around 90% in most of the cases and up to 97% when using the XG 1/3 catalyst. This time, the AG Al + 5Ni catalyst reached a selectivity of 80% at the highest temperature. In this case, in accordance with the previous results, the AG Al catalyst did not prove to be useful as a catalyst in the Sabatier reaction with a UV light source, as it obtained a maximum 34% CO_2_ conversion and 28% CH_4_ selectivity at 400 °C.

As can be seen in the activity results, xerogels exhibit, in general, better catalytic performances than the impregnated aerogels. In both types of materials, a higher Ni content usually results in a higher CO_2_ conversion, and compared to the poor activity of AG Al results, it is proven that nickel enhances the CO_2_ conversion and selectivity towards CH_4_ [69,70,71]. The high surface area allowed good particle dispersion, as seen in the TEM results, which also promoted the high activity of the catalysts. Additionally, as seen in the H_2_-TPR results, impregnated aerogels appear to have a prominent spinel structure, which is harder to reduce, perhaps explaining their lower catalytic activity.

As expected, higher temperatures promote higher CO_2_ conversion, usually reaching the maximum value at the highest temperature. Furthermore, both lights successfully enhanced the activity of the catalysts, in accordance with the absorbance observed in the UV-vis DRS results. The xerogel catalysts exhibited higher conversion values at low temperatures (300 and 350 °C) with both visible and UV lights. This behavior can be attributed to the broad absorption peaks found in both regions of the spectrum, as it was observed in the UV-vis DRS results. The enhancement of the catalytic activity could not be supported with the reaction mechanism, as it was not studied, although it is in accordance with the results of other works [72,73,74]. On the other hand, the impregnated aerogel catalysts did not exhibit any remarkable improvement in the catalytic activity with any of the light sources, as their absorption at any wavelength was poor.

Considering the benefits of using sol–gel as a synthesis method of catalysts, such as improved specific surface area and thus improved active metal dispersion, further studies exploring the viability of this method to synthesize catalysts for CO_2_ methanation should be performed. In one case, Tabarkhoon et al. synthesized NiO/Al_2_O_3_ xerogel-based catalysts and studied their catalytic activity in a range of temperatures between 250 and 550 °C [75]. The CO_2_ conversion of their catalyst increased with temperature and reached its maximum at 400 °C, where a value below 70% was obtained. This result is superior to the ones obtained by the impregnated aerogel catalysts but similar to our xerogel catalysts when no light source is employed. When enhancing thermal activity with light source irradiation, our xerogels present a higher activity at a lower temperature, which makes them more sustainable and affordable to use. In addition, the catalyst mass used in our experiments was 1/20 of the mass used in their experiments, demonstrating the high productivity of our materials.

When comparing our catalysts to noble metal-based catalysts, such as bimetallic Ru- and Rh- based catalysts impregnated in Al_2_O_3_ spheres at a 0.5% wt [76], it is very challenging to outperform the astonishing catalytic activity they present. These catalysts exhibit 80% CO_2_ conversion and 100% CH_4_ selectivity at just 320 °C, which differs significantly from the performance of our catalysts under the same conditions. Even if using either of our source lights could achieve comparable results, these catalysts remain highly attractive as they cost less due to using only thermal effects and requiring low active metal mass. However, an economic assessment should be performed when comparing these catalysts to our own, considering the low catalyst mass used in each experiment (5 mg) and the low price of nickel.

Additionally, it is important to note the high Ni dispersion in the samples, which affects both the surface properties, as mentioned before, and the catalytic activity. This observation is in agreement with the study by Daroughegi et al., in which CeO_2_-promoted Ni-Al_2_O_3_ catalysts were synthesized [77]. In this case, the addition of CeO_2_ increased the surface area, pore radius, and pore volume, enabling high and homogeneous distribution of the Ni particles; this resulted in smaller particle sizes and, thus, more active sites for methanation. As a result, CO_2_ conversions of nearly 80% were achieved at 350 °C, with a CH_4_ selectivity close to 100%.

## 3. Conclusions

This study focused on a direct comparison between two families of gel-based catalysts prepared via different routes to test the effect of the preparation route on their activity in the CO_2_ methanation reaction. The characterization of the catalysts revealed that the impregnated aerogels yielded surface areas as high as 209 m^2^/g, whereas the xerogels reached values of 276 m^2^/g. Through various techniques, such as XRD and UV-vis DRS, the presence of the metallic species in the catalysts was confirmed. It was concluded that both NiO and NiAl_2_O_4_ are present in the impregnated aerogels, while in the xerogels, NiO appears to have an overall higher importance, which was also confirmed by the H_2_-TPR results. Regarding the light-assisted CO_2_ methanation catalytic activity, it was observed that xerogels present an overall higher activity in comparison with the impregnated aerogels, which could be attributed to the prominence of the nickel spinel structure on the impregnated catalysts. As expected, the use of light sources enhanced the catalytic activity of the materials. When visible light was applied, CO_2_ conversions of 76% were reached at just 350 °C, compared to the 45% obtained in dark conditions. Moreover, UV light generally enhanced the catalytic activity more than visible light, as observed when comparing the results of the catalyst mentioned above. In this case, CO_2_ conversion reached values of 74% at the lower temperature (2091.7 mmol CH_4_·gcat^−1^·h^−1^), which is higher than the 68% achieved under a visible light source (1636.1 mmol CH_4_·gcat^−1^·h^−1^) and significantly higher than the 38% obtained when no light was used (170.2 mmol CH_4_·gcat^−1^·h^−1^). Additionally, CH_4_ selectivity was higher than 90% and close to 100% in most cases when using xerogels as catalysts, while the impregnated aerogels needed the use of light sources and higher temperatures to get close to these values.

Therefore, on the one hand, this comparison shows that the preparation route strongly affects Ni speciation and reducibility, which in turn governs the catalytic response under dark and light-irradiated conditions. On the other hand, the use of a simple drying method such as oven drying to obtain xerogels was successful in synthesizing catalysts with high nickel dispersion; these catalysts were highly active and showed a significant response under visible and UV irradiation. However, further research is needed to improve the surface area of the catalysts, which could further enhance the Ni dispersion and potentially promote catalytic activity.

## 4. Materials and Methods

### 4.1. Catalyst Preparation

Al aerogels were synthesized via the sol–gel method described by Gash et al. [78], employing propylene oxide as the gelation agent, followed by a CO_2_ supercritical drying of the gels and a subsequent impregnation with Ni. In the case of the Al/Ni xerogels, the gels were synthesized following the same method as the aerogels, but they were dried in an oven. The reagents needed for these synthesis were nickel (II) chloride hexahydrate (NiCl_2_·6H_2_O; 99.9% purity; Sigma-Aldrich, St. Louis, MO, USA) and aluminum nitrate (III) nonahydrate (Al(NO_3_)_3_·9H_2_O; >98% purity; Honeywell, Seelze, Germany) as metal precursors; liquid propylene oxide (PO; >99.5% purity; Sigma-Aldrich, Steinheim, Germany) as the gelation agent; and ethanol (EtOH; pure; PanReac, Darmstadt, Germany) as the solvent.

In the case of the aerogels, 1.758 g of the Al precursor was dissolved and stirred in 7.5 mL of ethanol until complete dissolution of the salt, followed by the slow addition of 3.6 mL of PO, in order to obtain a PO mol:precursor mol ratio of 11. The gel was aged for 24 h and washed several times with EtOH before supercritical drying. Multiple slices of the synthesized gel were deposited inside the dryer at a temperature of 10 °C and pressure of 1 bar, and it was subsequently filled with liquid CO_2_ (lCO_2_) until a pressure of 50 bar was reached. Next, several lCO_2_ exchanges were carried out to remove the remainder of EtOH, along with other impurities. Finally, the temperature on the system was elevated to 40 °C (70–80 bar), turning the lCO_2_ into supercritical CO_2_ (scCO_2_). The dryer was maintained at these conditions for 1 h before a slow rate depressurization. After obtaining the aerogel, it was calcined at 700 °C in air at a heating rate of 2 °C/min for 4 h. Afterwards, three different samples were obtained by wet impregnating the Al gels with NiCl_2_·6H_2_O at a constant pH of 9, corresponding to 5% wt, 10% wt, and 20% wt Ni loadings. As a final step, the impregnated aerogels were calcined again under the same conditions as the first calcination. The prepared samples were named AG Al + 5Ni, AG Al + 10Ni, and AG Al + 20Ni.

Regarding the Al/Ni xerogels, the metal precursors were dissolved in ethanol with three different Al:Ni molar proportions (1:3, 1:1, and 4:1) until the complete dissolution of the salts. Then, PO was slowly added to the solution. The volume of PO used varied across every sample to maintain the same PO mol/precursor mol ratio of 11, as in the case of the impregnated aerogels. The gel was once again aged for 24 h and cleaned with ethanol several times, and subsequently, it was dried in a muffle furnace at 80 °C at a rate of 2 °C for 16 h. Finally, the xerogels were calcined at 700 °C in air, at a heating rate of 2 °C/min, for 4 h.

### 4.2. Catalyst Characterization

The BET surface area (S_BET_) and various surface properties, such as the total pore volume (Vp), were calculated with the N_2_ adsorption and desorption isotherms (Autosorb iQ3; Quantachrome (Anton Paar), Madrid, Spain) after previous degasification at 250 °C.

X-ray diffraction (XRD) analysis was performed (X’pert PRO automatic diffractometer; Panalytical, San Sebastián de los Reyes, Spain) at 40 kV to determine the crystalline properties of the samples, applying a Cu-Kα radiation of λ = 1.5418 Å, with a scanning 2θ range of 5 to 80°. Additionally, the crystallite size was calculated with the Scherrer equation [79].

Inductively coupled plasma–optical emission spectroscopy (ICP-OES) experiments were carried out (Optima 2000 DV; Perkin Elmer, Tres Cantos, Spain) to determine the metal content of the samples. Before performing the analysis, they were digested in an acid solution. The samples were studied with a plasma flux of 15 L/min.

The morphology of the different samples was studied via transmission electron microscopy (TEM) (Talos F200X, 200 kV; Thermo Fisher, Alcobendas, Spain). The particle size distribution was measured with the help of the Fiji image processing package, included in the ImageJ2 software (version 2.17.0; National Institutes of Health, Bethesda, MD, USA). Additionally, the HAADF-EDX method was used for elemental mapping.

H_2_-temperature-programmed reduction (H_2_-TPR) experiments were used to study the reducibility behavior of the calcined catalysts (AutoChem II; Micromeritics, L’Hospitalet de Llobregat, Spain). The temperature was raised up to 850 °C at a rate of 10 °C/min, under a constant 5% H_2_/Ar flow, after drying the atmosphere with He at 200 °C for 30 min.

UV–visible diffuse reflectance spectroscopy (UV-vis DRS) was used to analyze the photochemical nature of the catalysts (V-770; Jasco, Madrid, Spain), measuring their absorbance at different wavelengths. Moreover, the energy band gap of each material was calculated with the experimental data of this technique. The diffuse reflectance of the samples was measured starting at 2200 nm and up to 200 nm, and a blank background correction was also performed.

X-ray photoelectron spectroscopy (XPS) analyses (5000 VersaProbe II; PHI (Irida), Madrid, Spain) were carried out to determine the oxidation states of the metals in the samples. A monochromatic X-ray source of Al (Kα, 1486.6 eV) and a beam diameter of 200.0 µm were used in these experiments. Furthermore, the surface Al/Ni ratios of the samples were calculated with this technique. An internal standard was used to correct the binding energy shift, which corresponded to the C 1 s signal at 284.8 eV.

### 4.3. Activity Tests

The catalytic activity tests were carried out in a photoreactor with an 8 mm open quartz window that is directly on top of the sample. This photoreactor has two different light sources available, with one of them emitting ultraviolet light (365 nm, 3.4 eV) and another one emitting visible light (470 nm, 2.7 eV). The intensity of the light was previously analyzed using a GL Optic Spectis 1.0 spectrometer, which was attached to an Opti Sphere 48 (GL Optic, Puszczykowo, Poland), and the selected light intensity was 2.4 W/cm^2^ for both light sources. The bench-scale plant (PID Eng&Tech, Alcobendas, Spain) had a maximum working temperature of 450 °C, which was controlled via a thermocouple placed inside the reactor, near the catalyst bed, and a maximum pressure of 10 bar. The chosen total gas flow for all tests was 50 mL_N_/min with a stoichiometric CO_2_:H_2_ = 1:4 gas composition. The composition of the effluent gas flow was analyzed with an online CompactGC 4.0 gas chromatograph (GAS, Breda, The Netherlands), which had a TCD and an FID detector.

Prior to each activity test, the catalyst particle size was fixed between 0.42 and 0.5 mm, and it was then reduced in a flow of 20% H_2_/N_2_. The H_2_-TPR results helped determine the reduction temperature for each material. Taking these results into account, a temperature of 700 °C was chosen to perform the reduction of the impregnated aerogels, at a heating rate of 5 °C/min and for 2 h. In the case of the xerogels, the reduction of the catalysts was carried out under the same conditions as the aerogels. The results of the activity tests were followed through a range of temperatures of 300 to 450 °C, and the pressure was fixed at 10 bar. In each test, a catalyst mass of 5 mg was used, enough to fill the small reactor. Additionally, a heating ramp of 5 °C/min was followed, and the system was maintained at each temperature for 1.5–2 h while the effluent gas composition was constantly analyzed.

The activity results, in terms of CO_2_ conversion and CH_4_ selectivity, were calculated using the following equations:

(4) $${CO}_{2}\;Conversion\;\left(\%\right)=\frac{{\left[{CO}_{2}\right]}_{in}-{\left[{CO}_{2}\right]}_{out}}{{\left[{CO}_{2}\right]}_{in}}\times 100$$

(5) $${CH}_{4}\;Selectivity\;(\%)=\frac{{\left[{CH}_{4}\right]}_{out}}{{\left[{CH}_{4}\right]}_{out}+{\left[CO\right]}_{out}}\times 100$$

## Figures and Tables

**Table 1:** Surface properties and Al/Ni molar ratios of prepared catalysts.

Sample	Nominal Al/Ni Molar Ratio	Al/Ni Molar Ratio	S_BET_ (m^2^/g)	V_t,pore_ (cm^3^/g)	D_BJH_ (nm)
AG Al	-	-	317 ± 31	1.88	19.1
AG Al + 5Ni	21.88	13.60	187 ± 16	0.27	3.5
AG Al + 10Ni	10.36	7.48	209 ± 19	0.25	3.7
AG Al + 20Ni	4.60	3.72	189 ± 17	0.33	4.2
XG 1/3	0.33	0.31	135 ± 11	0.29	6.3
XG 1/1	1.00	0.93	222 ± 20	0.34	5.4
XG 4/1	4.00	3.65	276 ± 26	0.35	4.2

**Table 2:** Band gap energies of the catalysts, calculated with the Tauc equation.

Sample	Band Gap Energy (eV)
AG Al	4.66
AG Al + 5Ni	5.05
AG Al + 10Ni	4.78
AG Al + 20Ni	3.48
XG 1/3	1.35
XG 1/1	2.82
XG 4/1	3.00

**Table 3:** Surface Al/Ni ratio of catalysts obtained via XPS.

Catalyst	Al/Ni Atomic Ratio
AG Al	-
AG Al + 5Ni	7.93
AG Al + 10Ni	5.76
AG Al + 20Ni	2.86
XG 1/3	0.45
XG 1/1	0.94
XG 4/1	3.16

## Data Availability

The data that support the findings of this study are available from the corresponding author upon reasonable request.

## Supplementary Materials

The following supporting information can be downloaded at https://www.mdpi.com/article/10.3390/gels12050420/s1, Figure S1: N_2_ adsorption–desorption isotherms of the catalysts: AG Al, AG Al + 5Ni, AG Al + 10Ni, AG Al + 20Ni, XG 1/3, XG 1/1, XG 4/1; Figure S2: TEM micrographs and EDX mapping of AG Al, AG Al + 5Ni, AG Al + 10Ni, XG 4/1; Figure S3: Tauc plots for all catalysts, used to calculate the direct band gap energy.

## Abbreviations

The following abbreviations are used in this manuscript:

EGD	European Green Deal
EU	European Union
PtG	Power-to-Gas
lCO_2_	Liquid CO_2_
scCO_2_	Supercritical CO_2_
RWGS	Reverse Water–Gas Shift
AG	Aerogel
XG	Xerogel
ICP-OES	Inductively Coupled Plasma–Optical Emission Spectroscopy
BET	Brunauer, Emmet, and Teller Theory
BJH	Barrett–Joyner–Halenda
TEM	Transmission Electron Microscopy
HAADF	High-Angle Annular Dark Field
EDX	Energy-Dispersive X-Ray Spectroscopy
XRD	X-Ray Diffraction
XPS	X-Ray Photoelectron Spectroscopy
UV-vis DRS	UV–Visible Diffuse Reflectance Spectroscopy
H_2_-TPR	H_2_ Temperature-Programmed Reduction
TCD	Thermal Conductivity Detector
FID	Flame Ionization Detector
