# Supplementary material for: Comparative Study of Ni-Impregnated Alumina Aerogels and Ni-Al Xerogels for Light-Irradiation-Assisted CO2 Methanation
Source: Gels. 2026 May 11;12(5):420. doi: 10.3390/gels12050420 (PMC13205539; doi:10.3390/gels12050420)
Supplement: Supplementary file 1 [file gels-12-00420-s001.zip › gels-4226262-supplementary.pdf]

Supplementary Materials

Comparative Study of Ni-Impregnated Alumina Aerogels and Ni-Al Xerogels for Light-Irradiation-Assisted CO_2 Methanation

Daniel Estevez, Haritz Etxeberria and Victoria Laura Barrio *

School of Engineering of Bilbao, University of the Basque Country (UPV/EHU), Plaza Ingeniero Torres Quevedo 1, 48013 Bilbao, Spain; daniel.estevez@ehu.eus (D.E.); haritz.echeverria@ehu.eus (H.E.)
* Correspondence: laura.barrio@ehu.eus

Supporting Figures

a)

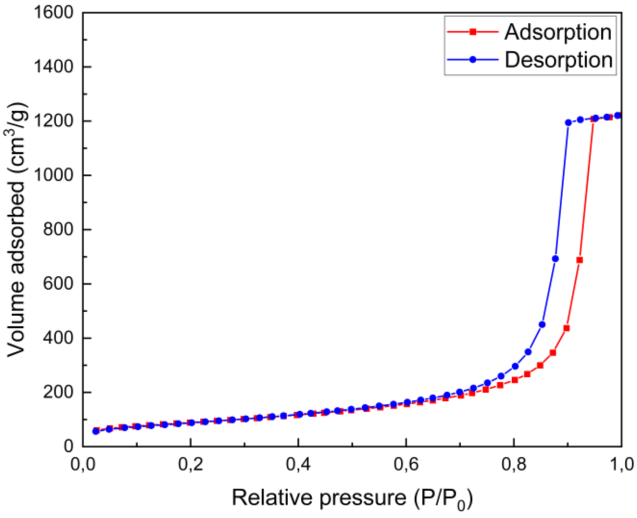

b)

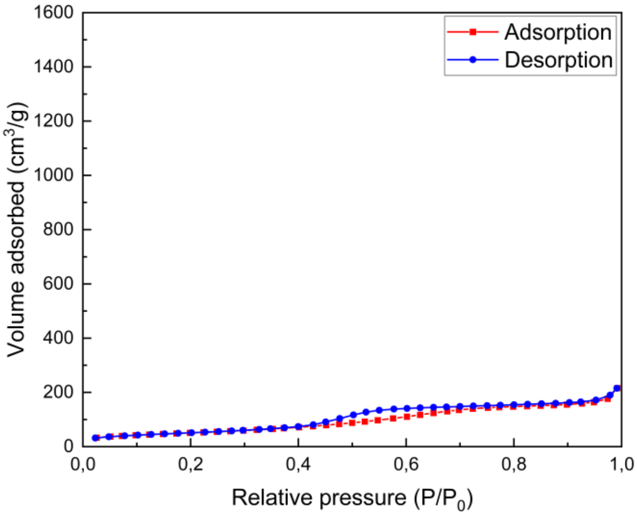

c)

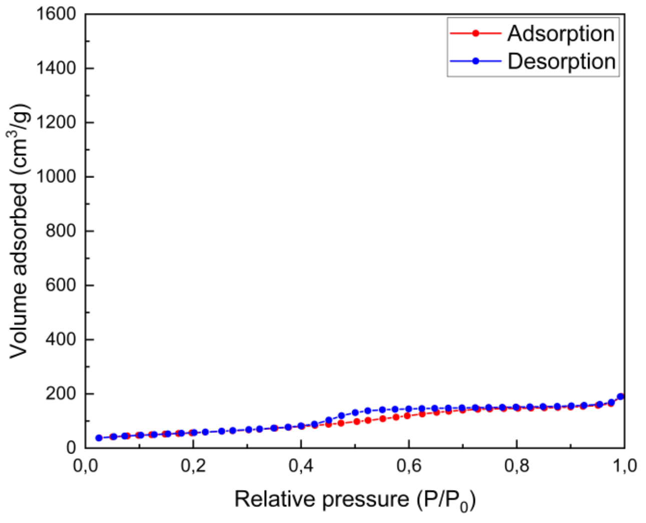
Adsorption
Desorption
Volume adsorbed (cm3/g)
Relative pressure (P/P0)
1600
1400
1200
1000
800
600
400
200
0
0,0
0,2
0,4
0,6
0,8
1,0

d)

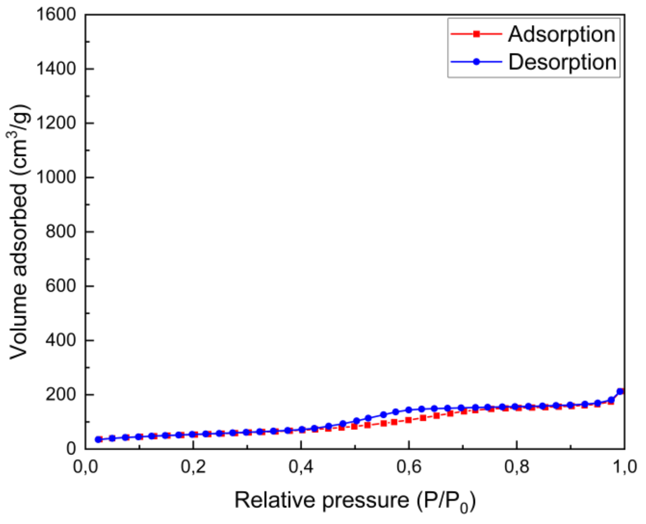
Adsorption
Desorption
Volume adsorbed (cm3/g)
Relative pressure (P/P0)
1600
1400
1200
1000
800
600
400
200
0
0,0
0,2
0,4
0,6
0,8
1,0

e)

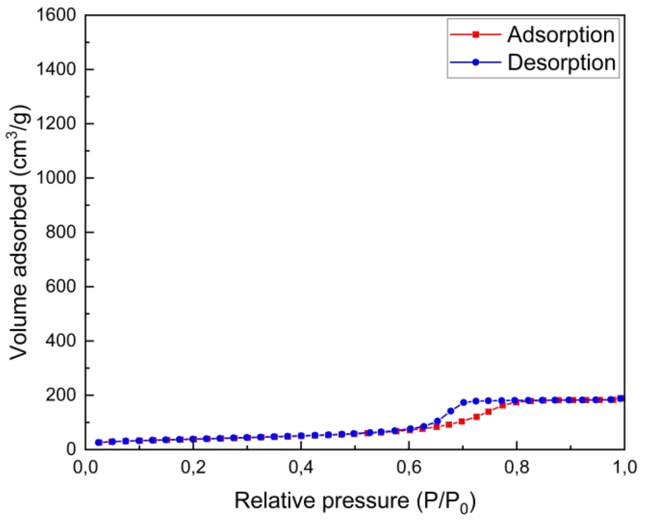
Adsorption
Desorption
Volume adsorbed (cm3/g)
Relative pressure (P/P0)
1600
1400
1200
1000
800
600
400
200
0
0,0
0,2
0,4
0,6
0,8
1,0

f)

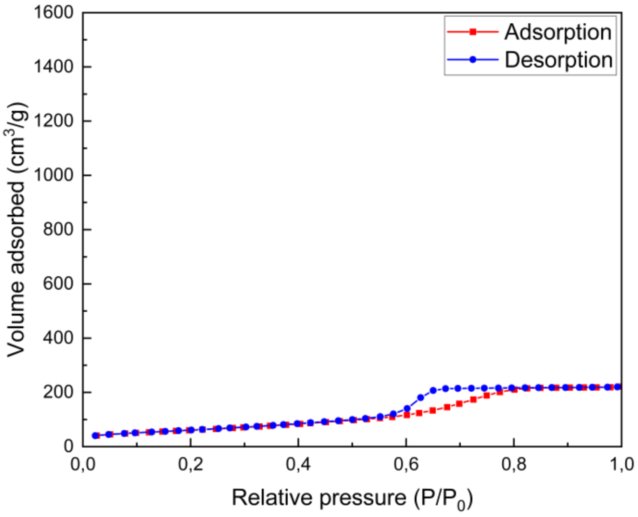

g)

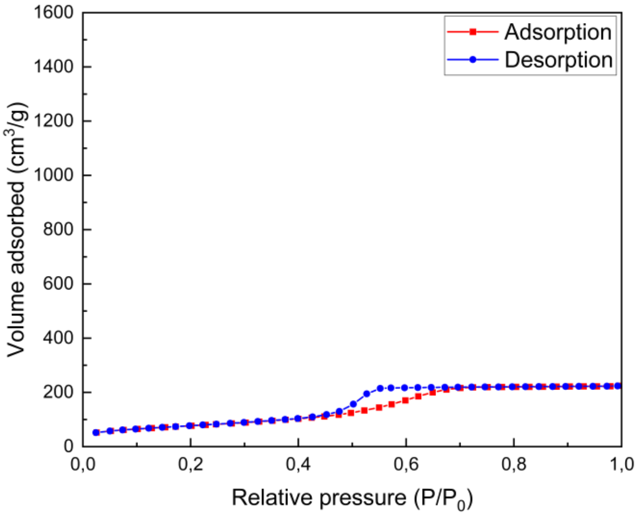

Figure S1. N_2 adsorption–desorption isotherms of the catalysts: (**a**) AG Al, (**b**) AG Al + 5Ni, (**c**) AG Al + 10Ni, (**d**) AG Al + 20Ni, (**e**) XG 1/3, (**f**) XG 1/1, (**g**) XG 4/1.

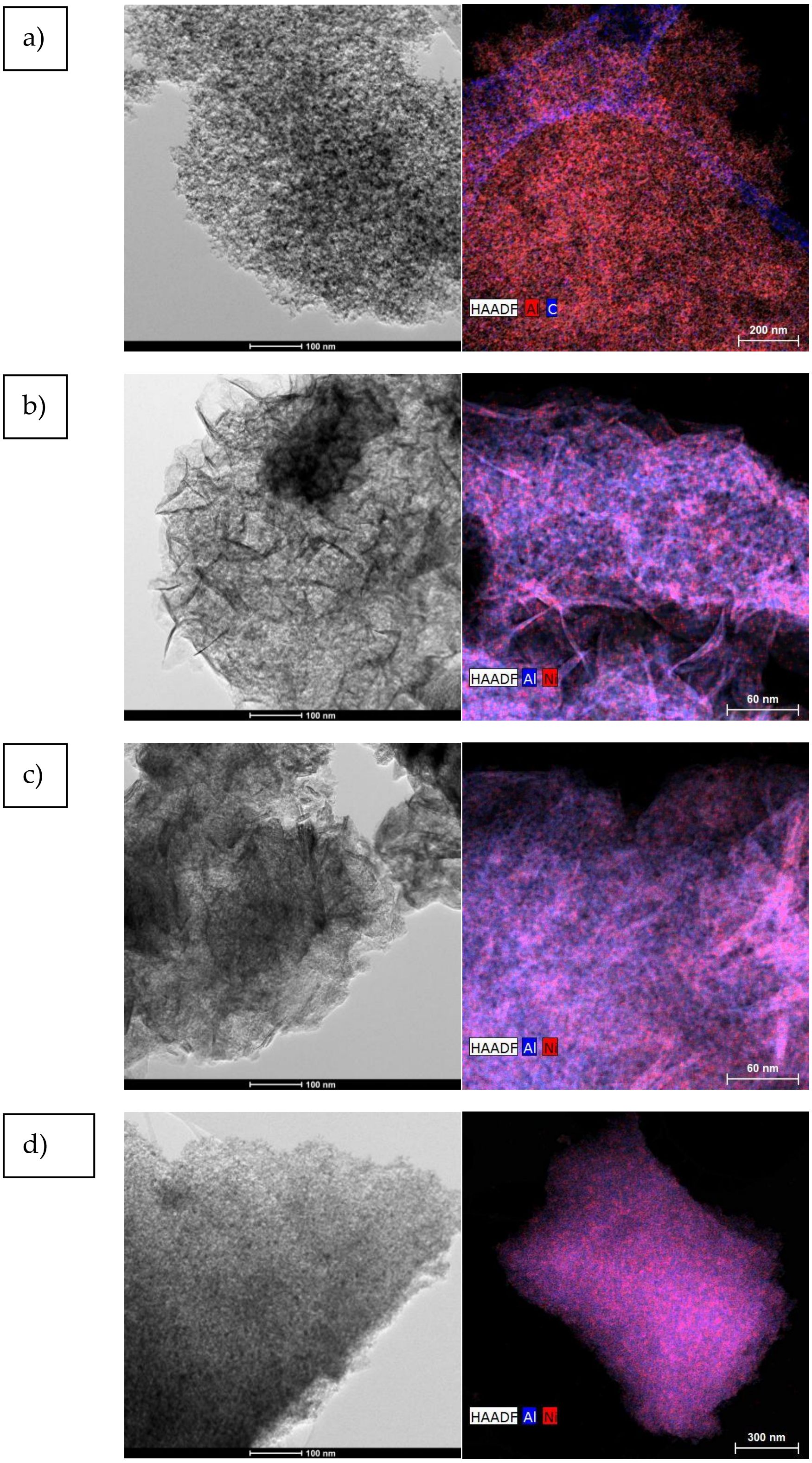

Figure S2. TEM micrographs (left) and EDX mapping (right) of (**a**) AG Al, (**b**) AG Al + 5Ni, and (**c**) AG Al + 10Ni, (**d**) XG 4/1.

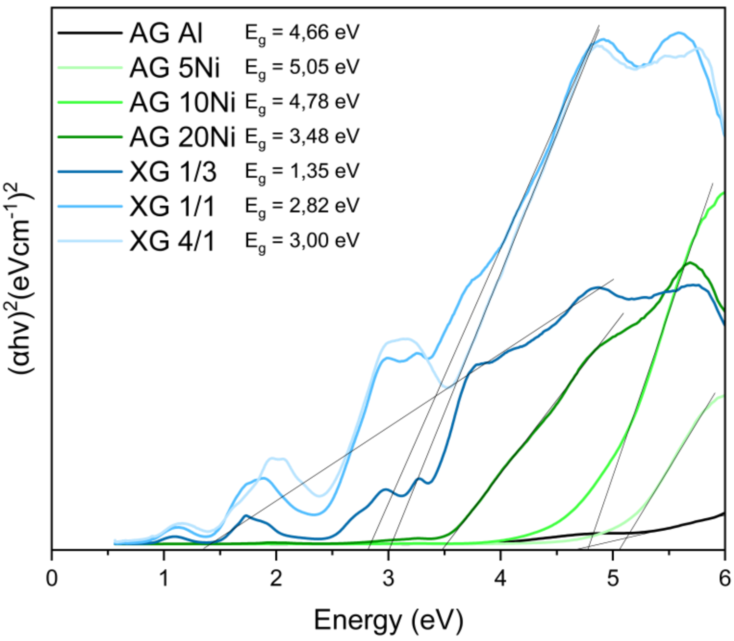

Figure S3. Tauc plots for all catalysts, as used to calculate the direct band gap energy.
